# Rac2-Deficiency Leads to Exacerbated and Protracted Colitis in Response to *Citrobacter rodentium* Infection

**DOI:** 10.1371/journal.pone.0061629

**Published:** 2013-04-16

**Authors:** Ramzi Fattouh, Cong-Hui Guo, Grace Y. Lam, Melanie G. Gareau, Bo-Yee Ngan, Michael Glogauer, Aleixo M. Muise, John H. Brumell

**Affiliations:** 1 Cell Biology Program, Hospital for Sick Children, Toronto, Ontario, Canada; 2 Department of Pathology, Hospital for Sick Children, Toronto, Ontario, Canada; 3 Faculty of Dentistry, University of Toronto, Toronto, Ontario, Canada; 4 Division of Gastroenterology, Hepatology, and Nutrition, Department of Pediatrics, Hospital for Sick Children, University of Toronto, Toronto, Ontario, Canada; 5 Institute of Medical Science, University of Toronto, Toronto, Ontario, Canada; 6 Sickkids IBD Centre, Hospital for Sick Children, Toronto, Ontario, Canada; 7 Department of Molecular Genetics, University of Toronto, Toronto, Ontario, Canada; McGill University, Canada

## Abstract

Recent genetic-based studies have implicated a number of immune-related genes in the pathogenesis of inflammatory bowel disease (IBD). Our recent genetic studies showed that *RAC2* is associated with human IBD; however, its role in disease pathogenesis is unclear. Given Rac2’s importance in various fundamental immune cell processes, we investigated whether a defect in Rac2 may impair host immune responses in the intestine and promote disease in the context of an infection-based (*Citrobacter rodentium*) model of colitis. In response to infection, Rac2^−/−^ mice showed i) worsened clinical symptoms (days 13–18), ii) increased crypt hyperplasia at days 11 and 22 (a time when crypt hyperplasia was largely resolved in wild-type mice; WT), and iii) marked mononuclear cell infiltration characterized by higher numbers of T (CD3^+^) cells (day 22), compared to WT-infected mice. Moreover, splenocytes harvested from infected Rac2^−/−^ mice and stimulated *in vitro* with *C. rodentium* lysate produced considerably higher levels of interferon-γ and interleukin-17A. The augmented responses observed in Rac2^−/−^ mice did not appear to stem from Rac2’s role in NADPH oxidase-driven reactive oxygen species production as no differences in crypt hyperplasia, nor inflammation, were observed in infected NOX2^−/−^ mice compared to WT. Collectively, our findings demonstrate that Rac2^−/−^ mice develop more severe disease when subjected to a *C. rodentium*-induced model of infectious colitis, and suggest that impaired Rac2 function may promote the development of IBD in humans.

## Introduction

Inflammatory bowel disease (IBD) is a family of chronic, relapsing and remitting, inflammatory disorders of the gastrointestinal tract [1], [2]. The available evidence suggests that IBD develops as a result of an exaggerated immune response directed against the host microbiota. Despite considerable research the precise etiology of these diseases remains largely uncertain [3]. As there is no cure, and treatments are geared primarily at alleviating intestinal inflammation and symptoms, there is an urgent need to identify the determinants that predispose the development of IBD. Improved understanding of the causes of IBD will undoubtedly aid in the development of more effective treatment strategies.

In addition to various environmental factors it is clear that genetic factors also influence susceptibility to IBD. Recent genetic based studies have identified greater than 100 genetic loci as being associated with the development of IBD [2], [4], [5]. Of the numerous genes implicated by these studies, many are known to be integrally involved in various aspects of the immune response including, pathogen recognition, cytokine/growth factor signalling, intestinal epithelial barrier function and defense, and lymphocyte activation, among others.

We have recently identified several single nucleotide polymorphisms (SNPs) in the gene encoding RAC2, a small guanosine triphosphatase (GTPase) that is almost exclusively expressed in leukocytes [6]. RAC2 is one of four regulatory cytosolic subunits (the other three being p40^phox^, p47^phox^, p67^phox^) that together with two transmembrane components gp91^phox^ (Nox2) and p22^phox^ constitute the predominant nicotinamide adenine dinucleotide phosphate (NADPH) oxidase (Nox) complex in leukocytes [7], [8]. The functional requirement for RAC2 in various immune cell-related processes has been explored in several *in vitro*-based studies using, primarily, macrophages and neutrophils; two cell types of the innate immune response that are critically required for protection against microbes. Collectively, previous findings demonstrate that in the absence of Rac2, macrophages and/or neutrophils display suppressed reactive oxygen species (ROS) production, defective chemotaxis, impaired phagocytosis, and decreased microbial killing [9], [10], [11], [12], [13], [14], [15], [16], [17], [18]. Importantly, the involvement of Rac2 in immunity is not limited to cells of the innate arm. Indeed, Rac2-deficiency has also been shown to impact B- and T-cell migration, activation, development (to a lesser extent in T-cells) [19], [20], [21], [22], [23], and, in some reports, T-cell differentiation into T-helper type 1 (Th1) cells [21], [24]. The contribution of Rac2 to host defense responses *in vivo* has been less well studied. Rac2^−/−^ mice infected intradermally with *Leishmania major* displayed little to no impaired ability to clear infection [21]. However, in a separate report Rac2^−/−^ mice challenged intravenously with the opportunistic pathogen *Aspergillus fumigatus*, showed marked increases in mortality, decreased survival time to death, and increased pathogen loads, compared to infected wild-type (WT) mice [13]. Interestingly, a dominant-negative mutation in RAC2 (D57N) has been described in two case reports of infants less than eight-weeks of age [25], [26], [27], [28]. Both patients suffered from repeated life-threatening bacterial infections necessitating stem cell transplantation. Together, the evidence from these *in vivo* reports further support the notion that impairments in RAC2 may compromise host defenses against, at least some, pathogens.

Whether defects in RAC2 impact the intestinal immune response, *in vivo*, and influence the development of IBD is unknown. Given the putative importance of RAC2 to immunity and the strong connection between altered immune functioning and IBD pathogenesis, we investigated the impact of defective Rac2 function on the intestinal immune response and the development of disease using a model of *Citrobacter rodentium*-induced colitis. Our findings show that Rac2^−/−^ mice infected with *C. rodentium* develop worsened symptoms, augmented and protracted colonic epithelial hyperplasia, and marked colitis associated with heightened T-cell responsiveness.

## Materials and Methods

### Ethics Statement

Experiments described in this study were carried out in accordance with the Guide for the Humane Use and Care of Laboratory Animals and were approved by the University of Toronto (Toronto, ON, Canada; #9558) and The Hospital for Sick Children’s Animal Care Committees (Toronto, ON, Canada; # 13639).

### Animals

Previously generated and characterized Rac2^−/−^ and wild-type (WT) mice (on a Balb/c.129Sv background) were bred as previously described at the University of Toronto Animal Care Facility [14], and maintained as separate lines. The genotypes of WT and Rac2^−/−^ mice were confirmed by PCR analysis on tail DNA as previously described [13]. C57BL/6 and gp91^phox−/−^ (Cybb/tm1d; Stock#2365) mice were purchased from The Jackson Laboratory (Bar Harbor, ME). All experiments were performed with 7–9 week old male mice that were maintained on a 12-hour light-dark cycle, with food and water available *ad libitum*.

### Infection

*C. rodentium*, strain DBS100 (ATCC #51459), was kindly provided by Dr. Philip Sherman (The Hospital for Sick Children in Toronto; originally from David Schauer, Massachusetts Institute of Technology). Bacteria were grown on Luria broth (LB) plates overnight at 37°C, followed by overnight culture in LB broth at 37°C without shaking. *C. rodentium* (3×10^8^ cfu) was administered to mice by orogastric gavage in 200 µl of LB broth. Vehicle control mice were given an equal volume of sterile LB broth.

### Collection and Measurement of Specimens

Mice were monitored every other day for weight change, stool consistency, rectal bleeding, and general appearance. At the indicated time points, colon (terminal 3 cm starting from ∼0.5 cm away from the anus) and spleen were collected. Following dissection, the lumen of the colon was washed, gently, with sterile PBS using a feeding needle. The terminal 0.75 cm of the colon was cut and fixed in 10% formalin for 24 hours for histochemical and morphometric analysis. Where applicable, the remaining ∼2.25 cm were collected in PBS for leukocyte isolation and flow cytometric analysis. Harvested spleens were placed in sterile tubes containing sterile Hank’s Balanced Salt Solution (HBSS) for splenocyte culture.

### Histological and Clinical Symptoms Scoring

Inflammatory infiltrate and goblet cell loss were scored by a pathologist blinded to the experimental conditions based on the scoring system used in [29] and shown in Table S1. The clinical symptoms score was adapted from [29] and calculated as shown in Table S2.

### Histology and Immunohistochemistry

After formalin fixation, the colon was cut cross-sectionally in half, yielding two pieces of tissue. Tissues were embedded in paraffin, and two separate 3-µm-thick cross-sections (>100 µm apart) were cut and stained with hematoxylin and eosin (H&E). Immunohistochemistry for Ki-67, *C. rodentium*, and CD3 were performed using the protocol previously described in detail [30], with the following changes: tissues were not incubated in permeabilization buffer, heat-induced epitope retrieval was performed using a vegetable steamer and slides were boiled (95–100°C) in sodium citrate buffer for 25–30 minutes, and tissues were incubated in block solution for 45–60 minutes. The following antibodies were used: rat anti-mouse Ki-67 (TEC-3; Dako; 1∶250), rabbit anti-*C. rodentium* (generated by the Sherman Lab, The Hospital for Sick Children, 1∶250), rabbit anti-CD3 (polyclonal, Sigma, 1∶2000), goat anti-rabbit AlexaFluor 488 and 568 (Molecular Probes, 1∶200) and donkey anti-rat Cy3 (Jackson ImmunoResearch Laboratories Inc, 1∶200).

### Morphometric Analysis

H&E stained sections were visualized using a Leica DMI 6000B microscope, Leica DFC420 camera and Leica Application Suite Advanced Fluorescence software (Leica, Toronto, ON). Crypt length was assessed by microscopy on coded sections by a single blinded observer. A minimum of 15, non-adjacent and well-orientated, crypts were measured per sample and used to determine the average crypt length/mouse. Images and morphometric analysis of Ki-67, *C. rodentium*, and CD3 were captured and analyzed using Volocity (v6.0; Perkin Elmer, Billerica, Massachusetts) via a Hamamatsu Orca R2 camera and Nikon Ti-E microscope. Multiple images were taken of the colon cross-section of each individual mouse (∼5–10 at 20× magnification, encompassing 95% of each section). For quantification of Ki-67^+^ cells in the crypts, the number of Ki-67^+^ cells/crypt was counted manually by a blinded observer; a minimum of 15 crypts were counted per sample. For quantification of colonic epithelial-associated *C. rodentium*, a ‘region of interest’ was drawn to encompass all of the colon tissue observable in a given image, being careful to exclude luminal contents. Within this ‘region of interest’, a colour intensity was selected that best captured the stain of interest and minimized or eliminated background staining. The total areas of both the ‘region of interest’ and the stained area were quantified and weighted averages were calculated for each individual mouse. The data shown represent the average percentage of the area of interest that is stained with *C. rodentium*. Similarly for CD3, a ‘region of interest’ was identified, the total area quantified, and Volocity used to determine the number of cells stained positive for CD3 (on the basis on signal intensity) within this region. The data shown represent the average number of CD3^+^ cells per unit of area.

### Colonic Cell Isolation and Flow Cytometric Analysis

Leukocytes of the lamina propria were isolated from the colon by mechanical digestion, using a method similar to that described in [31] with modification. Colon pieces (∼2.25 cm) were, first, cut longitudinally, and then cross-sectionally in pieces of ∼0.5 cm in length. Tissues were then incubated in 5 ml Ca^2+^/Mg^2+^ free HBSS supplemented with 10 mM Hepes, 5 mM ethylenediamine tetra-acetic acid, and 1 mM dithiothreitol, pH 7.4 for 20 minutes at 37°C in a shaker. Tissues were then vortexed vigorously for ∼5 s and dissociated cells in the supernatant (predominantly epithelial) were separated by pouring the mixture through a 100 µm cell strainer (BD PharMingen, Mississauga, ON). Tissue pieces were returned to a fresh 5 ml aliquot of HBSS solution and the process was repeated for a total of four times. Tissues were then washed with FACS buffer (0.5% bovine serum albumin in PBS) to remove traces of EDTA, chopped into 1–2 mm pieces and incubated in RPMI supplemented with 25 mM Hepes and 0.125 Wunsch units/ml Liberase-TM (Roche, Laval, Quebec) for approximately 50–60 minutes at 37°C with shaking. The resulting tissue fragments and supernatant were poured through a 40 µm cell strainer, crushed with the plunger of a 3 ml syringe and rinsed with FACS Buffer. Cells were subsequently pelleted (1400 rpm for 10 minutes at 4°C), washed once with FACS buffer, and resuspended in a final volume of 0.5–1 ml. Total cell counts were then performed by trypan blue counting. Each sample was filtered again through a 40 µm cell strainer prior to surface marker staining. For staining, cells were first incubated with FcBlock (anti-CD16/CD32; BD PharMingen, Mississauga, ON, Canada) in FACS buffer for 15 minutes at 4°C (to minimize nonspecific binding). Antibodies were titrated to determine optimal concentration. For each antibody combination, 2×10^6^ cells were incubated with mAbs on ice for 30 minutes. Cells were then washed twice with FACS buffer, fixed in 2.5% paraformaldehyde for 20 minutes on ice, and washed once more. Data were collected using a LSRII™ (BD) flow cytometer and analyzed using FlowJo™ software (Tree Star Inc., Ashland, OR, USA). The following antibodies were used: anti-CD4 (fluoresceine-isothiocyanate-conjugated RM4-5 clone), anti-CD45 (Alexa-Fluor 700-conjugated 30-F11 clone), purchased from BD PharMingen; anti-CD3 (eFluor450-conjugated 500A2 clone), purchased from ebioscience (San Diego, CA, USA). The following gating strategy was used: whole leukocytes were first selected on the basis of CD45 expression, then ‘singlets’ were identified using FSC-W vs. FSC-A. CD4^+^ and CD8^+^ T cells were then identified by gating on the lymphocyte area on a graph of SSC vs FSC-A, and subsequently gating on CD4^+^ or CD8^+^ cells within the CD3^+^ population of the lymphocyte region. The ‘absolute cell numbers’ were calculated by multiplying the appropriate population percentages by the total cell count for each sample.

### Splenocyte Culture

Splenocytes were isolated by triturating whole spleens through a 40 µm cell strainer (BD), using the plunger from a 3 ml syringe, into HBSS. Cells were then centrifuged at 1200 rpm for 10 minutes at 4°C and red blood cells were lysed using ACK lysis buffer. HBSS was used to stop lysis after 1 minute (1∶10 volume ratio). Splenocytes were then washed in complete RPMI (RPMI supplemented with 10% FBS – Sigma Aldrich; 1% L-glutamine; 1% penicillin/streptomycin – Invitrogen, Grand Island, NY, USA; and 0.1% β-mercaptoethanol – Invitrogen). Splenocytes were then resuspended in complete RPMI at a concentration of 6×10^6^ cells/ml and cultured in medium alone, or with medium supplemented with a *C. rodentium* lysate (7.5 µg/ml; prepared by repeated rounds of freeze-thaw and sonication of live *C. rodentium*) in a round-bottom, 96-well plate (BD), in triplicate; 100 µl of medium ± lysate +100 µl of cell suspension per well. After 3 days of culture, supernatants were harvested and triplicates were pooled and stored at −20°C for cytokine measurements.

### Cytokine Measurements by ELISA

Levels of mouse interleukin (IL)-4, -17A and interferon (IFN)-**γ** were measured by ELISA using DuoSet kits purchased from R&D Systems (Minneapolis, MN, USA) and performed according to the manufacturer’s instructions. Each of these assays has a threshold of detection between 2–10 pg/ml.

### Data Analysis

Data were analyzed using SigmaStat v3.0 (SPSS Inc., Chicago, IL). Data are expressed as mean ± SEM. Results were analyzed using two-way analysis of variance (ANOVA) with Tukey’s post hoc test. Differences were considered statistically significant at P<0.05.

## Results

### *C. rodentium* Infection Induces Increased and Prolonged Colonic Hyperplasia in Rac2^−/−^ Mice

**Figure 1:**
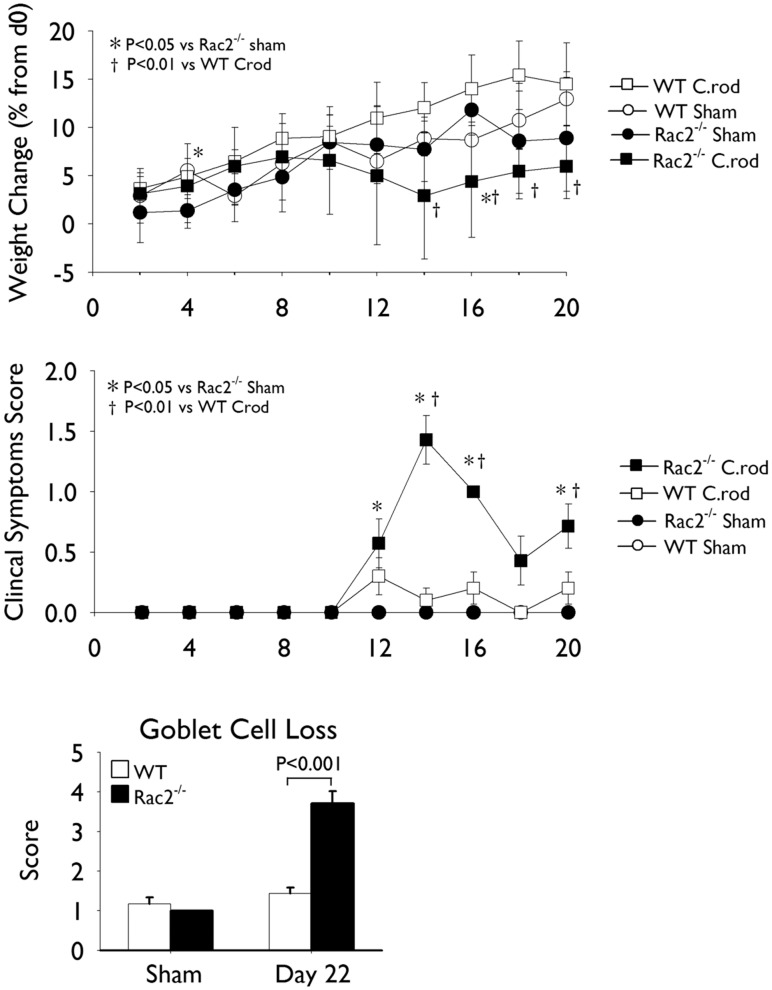
Rac2^−/−^ mice infected with Citrobacter rodentium display worsened signs of disease. Separate groups of wild-type (WT; *white bars/symbols*) and Rac2^−/−^ (*black bars/symbols*) mice were infected with *C. rodentium* or given vehicle control (sham) by orogastric gavage. (A) Percent weight change (relative to day 0) and (B) clinical symptoms score in WT and Rac2^−/−^ uninfected (*circles*) and infected (*squares*) mice. (C) Scoring of goblet cell loss at day 22 of infection. n = 4–11/group. Data are expressed as mean ± SEM and are from one of three independent experiments. Statistical analysis was performed using two-way ANOVA with Tukey’s post hoc test.

**Figure 2:**
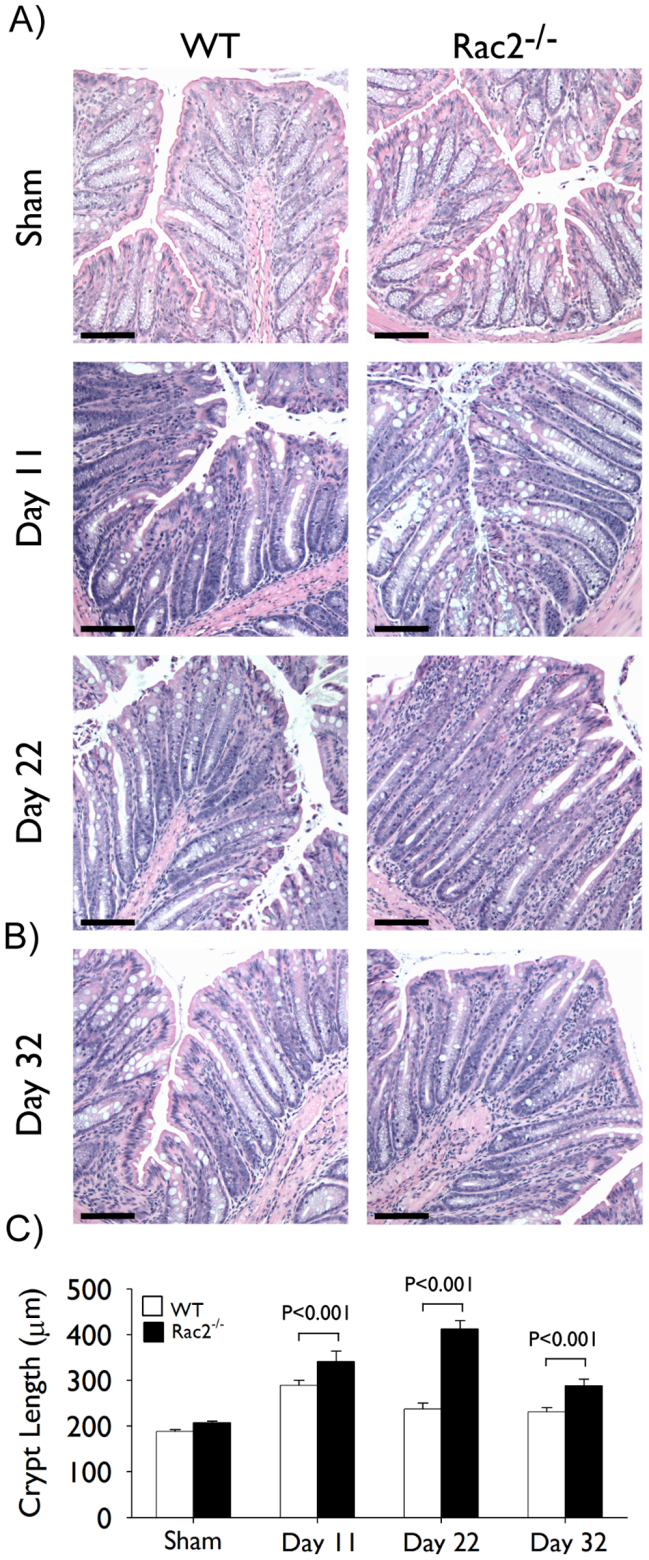
Kinetic analysis of Citrobacter rodentium-induced colonic hyperplasia in Rac2^−/−^ and WT mice. Separate groups of wild-type (WT; *white bars*) and Rac2^−/−^ (*black bars*) mice were infected with *C. rodentium* or given vehicle control (sham) by orogastric gavage. (A-B) Pictures show representative light photomicrographs of hematoxylin and eosin stained paraffin-embedded cross-sections of colon tissue obtained at the indicated time points of infection. Scale bar 100 µm. (C) Morphometric analysis of colonic hyperplasia; data represent average crypt length. All pictures were taken at 10× original magnification. n = 4–10/group. Data are expressed as mean ± SEM and are from one of three (in *A*) or two (in *B*) independent experiments. Statistical analysis was performed using two-way ANOVA with Tukey’s post hoc test.

To investigate the impact of a Rac2-deficiency on intestinal immune responses and the development of colitis, we subjected WT and Rac2^−/−^ mice to a model of *C. rodentium* induced colitis. Separate groups of mice were infected (day1) with *C. rodentium* by oral gavage and sacrificed on days 11 and 22 of infection, time points that represent peak and resolution stages of infection, respectively. In response to infection, Rac2^−/−^ mice displayed weight loss, a worsened clinical symptoms score (days 12–18), and considerable goblet cell loss (day 22; Figure 1) compared to infected WT mice. As expected, we observed that colonic hyperplasia was significantly increased in WT infected mice compared to uninfected (sham) controls at day 11, and that this response had largely resolved by day 22 (Figures 2A and C). Interestingly, Rac2^−/−^ infected mice developed significantly elevated hyperplasia at day 11 compared to WT mice that was considerably increased at day 22 (Figures 2A and C). To examine whether the colonic hyperplasia would continue to increase in Rac2^−/−^ mice we infected an independent cohort of mice and measured colonic hyperplasia at day 32 of infection. Although colonic hyperplasia remained significantly elevated over uninfected and WT infected mice at day 32, this response was decreased relative to day 22, indicating that the hyperplasia was resolving (Figures 2B and C).

### Colonic Hyperplasia is Driven by Enhanced Epithelial Cell Proliferation

**Figure 3:**
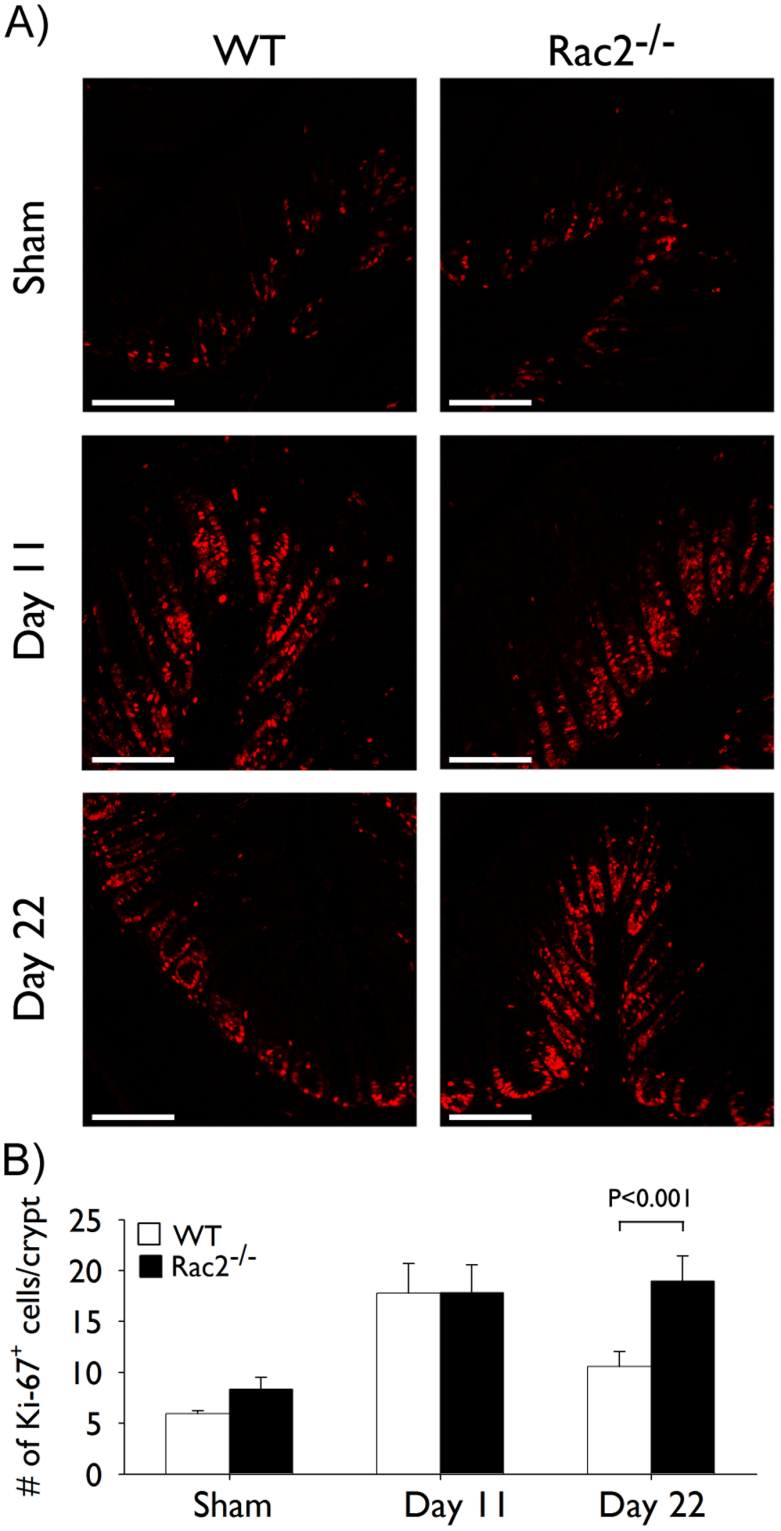
Prolonged colonic hyperplasia in Rac2^−/−^ mice develops due to continued epithelial cell proliferation. Separate groups of wild-type (WT; *white bars*) and Rac2^−/−^ (*black bars*) mice were infected with *Citrobacter rodentium* or given vehicle control (sham) by orogastric gavage. (A) Immunofluorescence for Ki-67. Pictures show representative images of Ki-67 stained paraffin-embedded cross-sections of colon tissue obtained at the indicated time points of infection. Scale bar 100 µm. (B) Morphometric analysis of Ki-67 staining; data represent the average number of Ki-67^+^ cells/crypt. All pictures were taken at 20× original magnification. n = 5–7/group. Data are expressed as mean ± SEM and are from one of two independent experiments. Statistical analysis was performed using two-way ANOVA with Tukey’s post hoc test.

To determine if the increase in colonic hyperplasia observed at day 22 in Rac2^−/−^ mice was mediated by active epithelial cell proliferation we quantified Ki-67 staining, a marker of proliferating cells, in the colon. We observed significant increases in the number of Ki-67^+^ epithelial cells/crypt that were similar between WT and Rac2^−/−^ infected mice at day 11 of infection (Figure 3). At day 22, the number of Ki-67^+^ cells had decreased substantially in WT infected mice but remained elevated in Rac2^−/−^ infected with *C. rodentium* (Figure 3), demonstrating that the increase in colonic hyperplasia in Rac2^−/−^ mice at day 22 is, at least in part, being driven by increased epithelial cell proliferation.

### Epithelial-associated *C. rodentium* is Cleared by day 22 of Infection in WT and Rac2^−/−^ Mice

**Figure 4:**
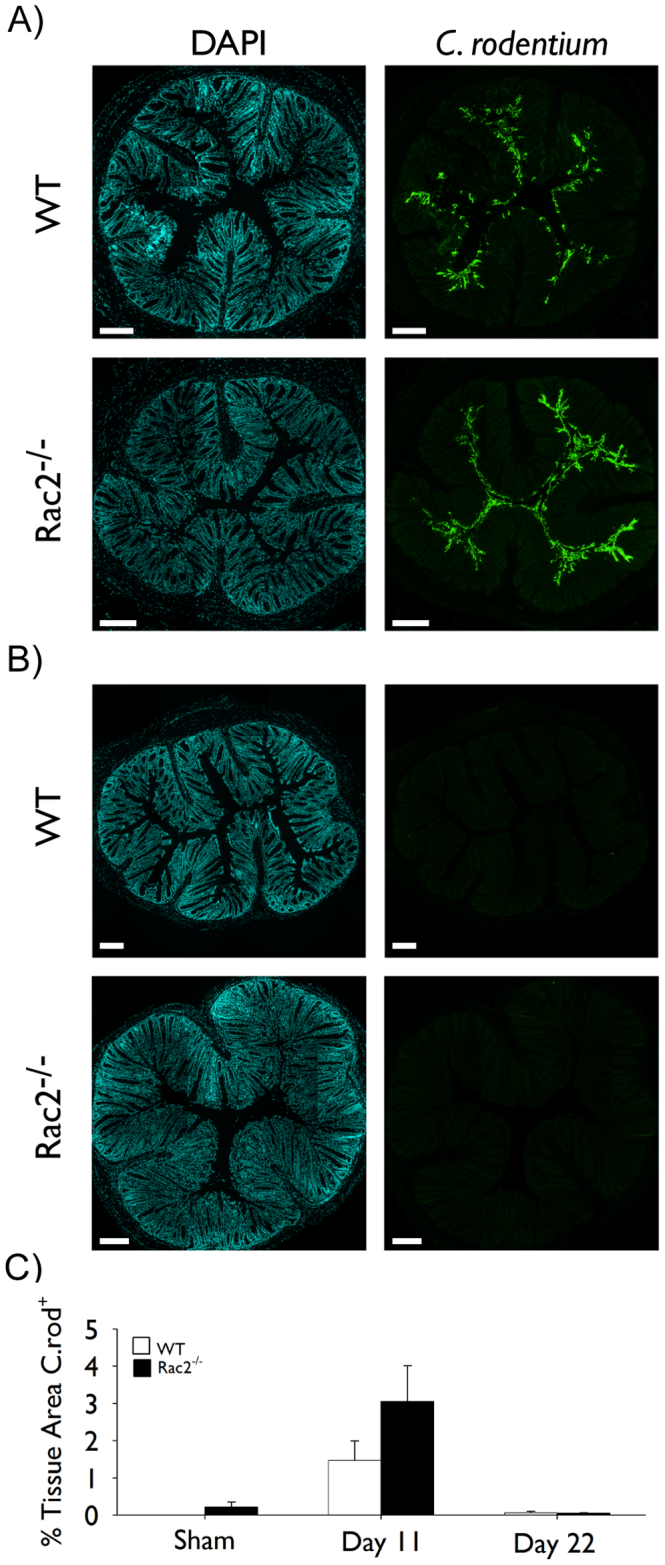
Epithelial-associated Citrobacter rodentium is cleared by WT and Rac2^−/−^mice at day 22 of infection. Separate groups of wild-type (WT; *white bars*) and Rac2^−/−^ (*black bars*) mice were infected with *C. rodentium* or given vehicle control (sham) by orogastric gavage. (A-B) Immunofluorescence for epithelial cell-associated *C. rodentium*. Pictures show representative stitched images of DAPI and *C. rodentium* co-stained paraffin-embedded cross-sections of colon tissue obtained at days 11 *(A)* and 22 *(B)* of infection. Scale bar 200 µm. (C) Morphometric analysis of *C. rodentium* staining; data represent the percentage of the total tissue area that is stained positive for *C. rodentium*. Individual images were taken at 10× and stitched together using image analysis software to create a composite cross-section. n = 6–8/group. Data are expressed as mean ± SEM and are from one of two independent experiments. Statistical analysis was performed using two-way ANOVA with Tukey’s post hoc test.

Given the putative importance of Rac2 in various immune cell-related functions and our observations of increased colonic hyperplasia, we questioned whether a deficiency in Rac2 negatively impacted bacterial clearance *in vivo*. To this end, we stained colon sections for the presence of *C. rodentium* and quantified the amount of bacteria associated with the colonic epithelium. Immunofluorescence analysis revealed a trend towards increased *C. rodentium* presence in Rac2^−/−^ infected mice compared to WT controls at day 11, but this difference was not statistically significant (Figures 4A and C). Moreover, no *C. rodentium* could be detected in either strain at day 22 of infection (Figures 4B and C). No *C. rodentium* was ever detected in uninfected mice (Figure 4C). These results suggest that Rac2^−/−^ mice are able to control and clear the infection.

### *C. rodentium* Infection Induces Marked Colitis in Rac2^−/−^ Mice

**Figure 5:**
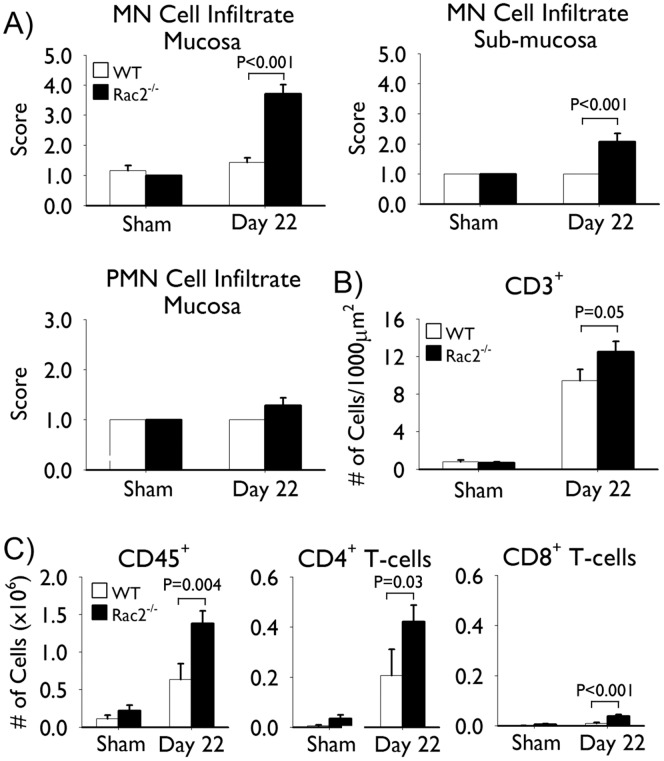
Rac2^−/−^ mice infected with Citrobacter rodentium develop marked mononuclear cell infiltration at day 22 of infection. Separate groups of wild-type (WT; *white bars*) and Rac2^−/−^ (*black bars*) mice were infected with *C. rodentium* or given vehicle control (sham) by orogastric gavage. (A) Scoring of mononuclear and polymorphonuclear cell infiltration into the submucosa and/or mucosa at day 22 of infection. (B) Average number of CD3^+^ cells per crypt in colon tissues obtained at day 22 of infection. (C) Flow cytometric analysis of colon digests showing the number of total leukocytes (CD45^+^ cells), T-helper (CD45^+^ CD3^+^ CD4^+^), and cytotoxic (CD45^+^ CD3^+^ CD8^+^) T-cells. n = 3–8/group in (*A-B*) and n = 3–4/group in (*C*). Data are expressed as mean ± SEM and are from one of two independent experiments. Statistical analysis was performed using two-way ANOVA with Tukey’s post hoc test.

Histopathological analysis revealed marked inflammation in the colons of Rac2^−/−^, but not WT, mice at day 22 of infection. Scoring of the inflammatory infiltrate by a pathologist blinded to the experimental conditions indicated that the inflammation was mononuclear cell in nature, with few if any polymorphonuclear cells observable at this stage of infection (Figure 5A). Immunofluorescence staining for CD3 demonstrated that a large number of these infiltrating cells were CD3^+^ (Figure 5B). To better quantify the extent of T-cell infiltrate we isolated cells from the colon and performed flow cytometric analysis to determine the number of, Th (CD45^+^ CD3^+^ CD4^+^) and cytotoxic T-cells (CD45^+^ CD3^+^ CD8^+^). In agreement with the histopathology, we isolated considerably higher numbers of total leukocytes (CD45^+^) and detected significantly more T-cells in the colons of infected Rac2^−/−^ mice compared to WT-infected mice (Figure 5C). The T-cell profile consisted predominantly of CD4^+^ T-cells with a limited number of CD8^+^ T-cells observed (Figure 5C).

### Rac2^−/−^ Mice Infected with *C. rodentium* Develop Heightened Th1 and Th17-associated Cytokine Responses

**Figure 6:**
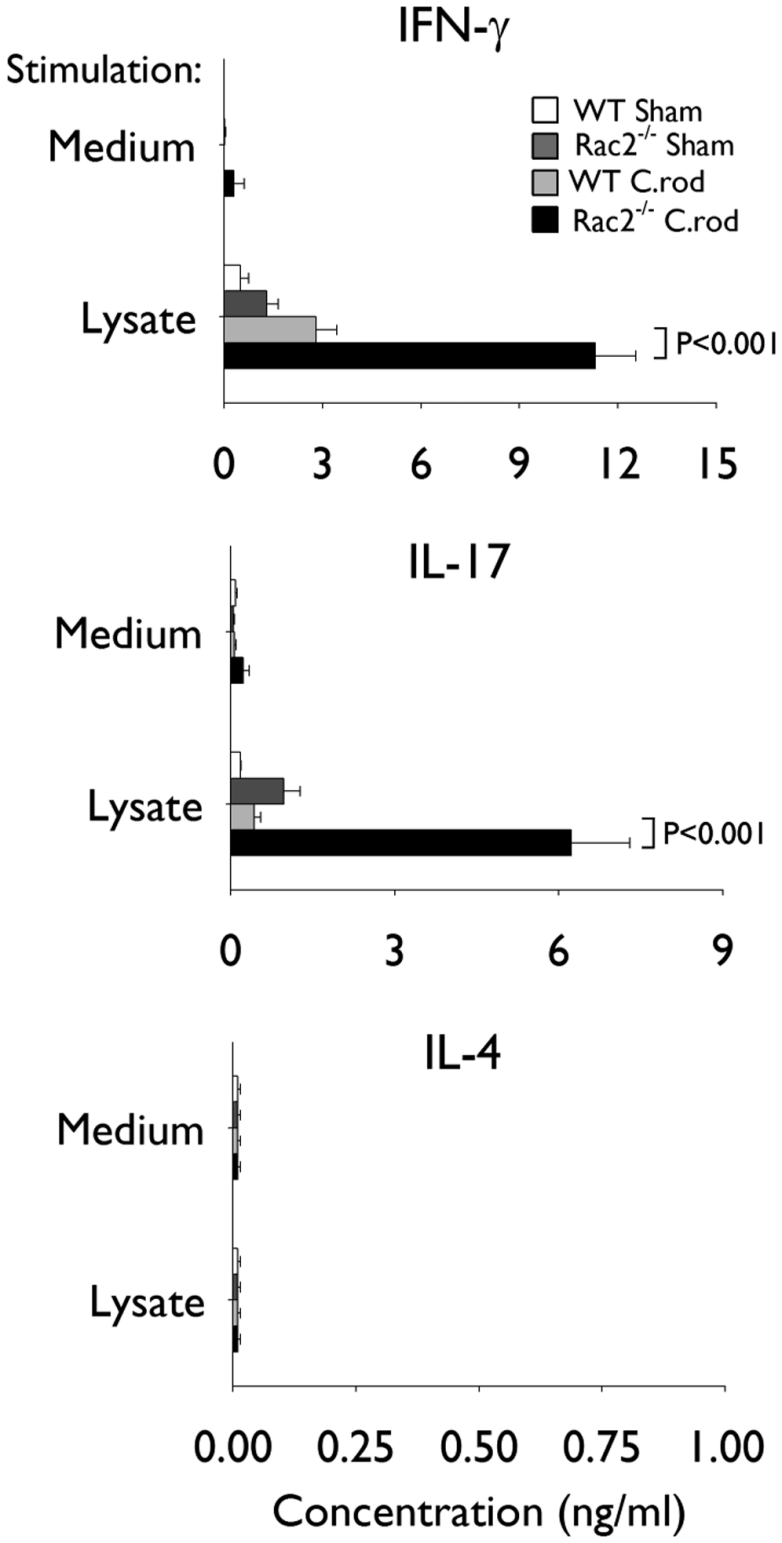
Heightened Th1- and Th17-associated cytokine production by splenocytes from Rac2^−/−^ mice at day 22 of infection. Separate groups of wild-type (WT) and Rac2^−/−^ mice were infected with *Citrobacter rodentium* or given vehicle control (sham) by orogastric gavage. Graphs show interferon-γ (IFN-γ), interleukin (IL)-17A and IL-4 production by splenocytes. Splenocytes from individual mice were harvested at day 22 of infection and cultured in medium alone or stimulated with *C. rodentium* lysate *in vitro*. n = 3–5/group. Statistical analysis was performed using two-way ANOVA with Tukey’s post hoc test. WT uninfected (*white bars*), WT *C. rodentium*-infected (*light grey bars*), Rac2^−/−^ uninfected (*dark grey bars*), and Rac2^−/−^ *C. rodentium*-infected (*black bars*).

To evaluate the impact of Rac2 deficiency on the T cell response against *C. rodentium* we harvested splenocytes from Rac2^−/−^ and WT-infected mice and measured the production of T cell-associated cytokines by ELISA following *in vitro* stimulation with a *C. rodentium* lysate. Splenocytes from infected Rac2^−/−^ mice produced considerably more of the Th1- and Th17-associated cytokines IFN-γ and IL-17A in response to *C. rodentium* than WT mice (Figure 6). No differences in IL-4 production (a Th2-associated cytokine) were observed between WT- and Rac2^−/−^infected mice. The proportion of Th cells in the spleen were similar between WT and Rac2^−/−^ mice, indicating that the differences in IFN-γ and IL-17A production observed were not due, simply, to an aberrant, overall, increase in baseline Th cell number resulting from genetic ablation of Rac2 (data not shown).

### Nox2^−/−^ Mice do not Develop Exaggerated Colonic Hyperplasia or Colitis following Infection with *C. rodentium*

**Figure 7:**
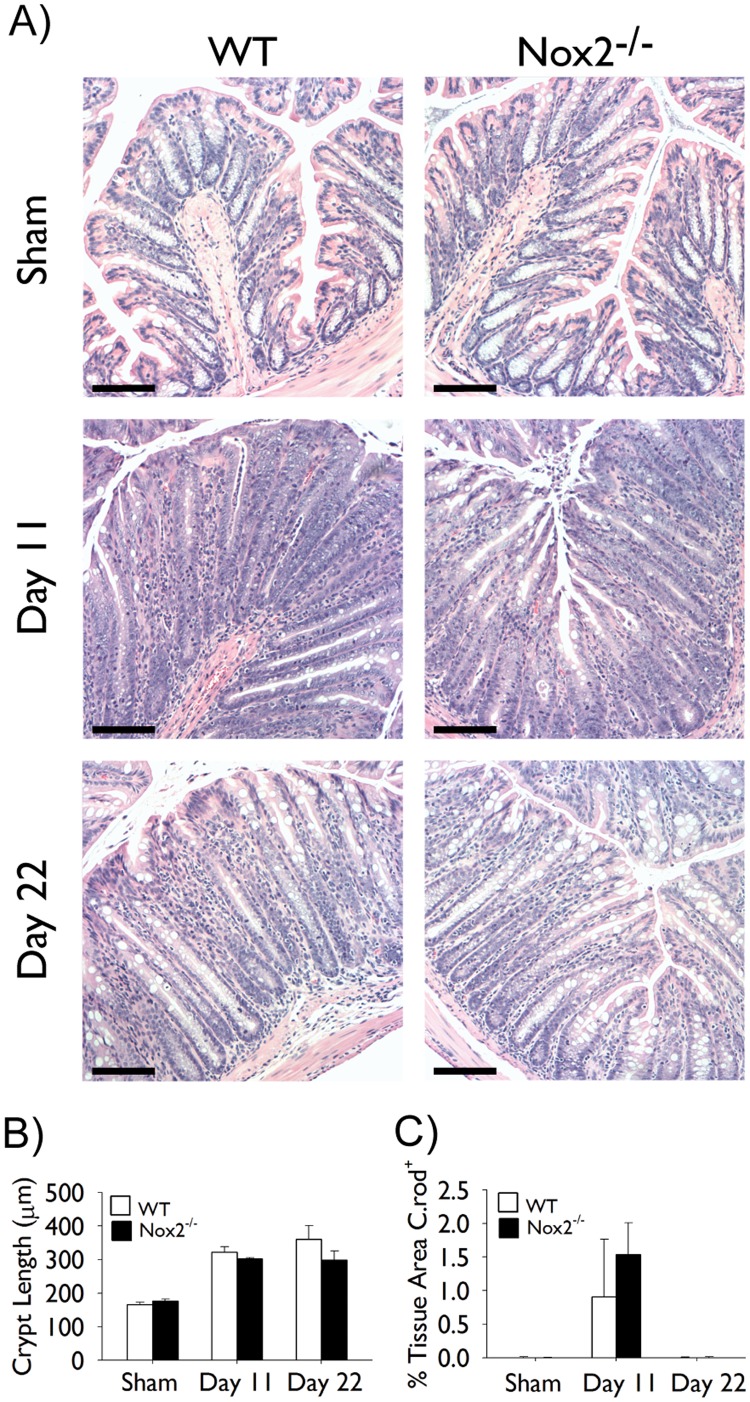
Nox2^−/−^ mice infected with Citrobacter rodentium do not develop exaggerated colonic hyperplasia or colitis. Separate groups of wild-type (WT; *white bars*) and Nox2^−/−^ (*black bars*) mice were infected with *C. rodentium* or given vehicle control (sham) by orogastric gavage. (A) Pictures show representative light photomicrographs of hematoxylin and eosin stained paraffin-embedded cross-sections of colon tissue obtained at the indicated time points of infection. Scale bar 100 µm. (B) Morphometric analysis of colonic hyperplasia at the indicated time points; data represent average crypt length. (C) Morphometric analysis of *C. rodentium* staining at the indicated time points of infection; data represent the percentage of the total tissue area that is stained positive for *C. rodentium*. All pictures were taken at 10× original magnification. n = 5–6/group. Data are expressed as mean ± SEM. Statistical analysis was performed using two-way ANOVA with Tukey’s post hoc test.

In light of the importance of Rac2 in NADPH oxidase-driven ROS production in leukocytes [7], [8], we questioned the extent to which the enhanced responses observed in infected Rac2^−/−^ mice were related to defects in leukocyte-derived ROS production. To address this, Nox2^−/−^ mice were infected with *C. rodentium* and colonic hyperplasia was examined at days 11 and 22 of infection. We observed that crypt hyperplasia developed to a similar extent in infected WT and Nox2^−/−^ mice (Figure 7A and B). Moreover, no significant differences were observed in the extent of *C. rodentium* colonization at day 11 of infection and bacteria were cleared from WT and Nox2^−/−^ mice at day 22 of infection (Figure 7C).

## Discussion

Our findings demonstrate that a genetic deficiency in Rac2 leads to worsened disease in mice infected with *C. rodentium* as evidenced by increased clinical symptoms score, colonic epithelial hyperplasia and colitis. However, the infection was not severe and all Rac2^−/−^ mice recovered from infection. In addition, Rac2^−/−^ mice did not appear to suffer from major innate immune defects as might have been predicted on the basis of the findings from previous *in vitro*-based studies [9], [10], [11], [12], [13], [14], [15], [16], [17], [18]. We noted that *C. rodentium* colonization was controlled and that all bacteria were cleared at day 22. Moreover, there was no evidence of systemic bacterial dissemination and no signs of colonic hemorrhaging. A previous study examining the impact of Rac2-deficiency on susceptibility to *L. major* infection reported that intradermally-infected Rac2^−/−^ mice displayed no differences in disease (i.e. lesion score and pathogen burden) compared to WT-infected mice [21]. Yet, in another report, Rac2^−/−^ mice were found to be more susceptible to systemic challenge with *A. fumigatus*, showing increased pathogen burden and mortality [13]. These findings, in conjunction with our own, suggest that the requirement for Rac2 in the host defense response against pathogens may depend heavily on the pathogen itself and the mode of infection.

How can our findings be reconciled with the findings from human and *in vitro* studies that suggest that the absence of Rac2 may exert a crippling effect on several aspects of the innate immune response, in particular, on immune phagocyte function? With respect to the findings of the two human case reports, interpretation of those results, as it pertains to RAC2, is somewhat confounded by the knowledge that the mutation identified therein is known to impart dominant-negative activity on RAC2 [26]. Thus, the observed defects in host defense responses in those patients may have developed, in part, from consequences unrelated to impaired RAC2 function per se. For example, the acquisition of dominant negative activity may result in the sequestration of guanine nucleotide exchange factors (GEFs) that activate other Rho GTPases potentially involved in distinct immune-related signalling pathways [32]. Furthermore, it is worth noting that in humans, RAC2 constitutes 75–95% of the total Rac content, whereas Rac1 and Rac2 are present in roughly equal proportions in murine neutrophils [12], [33], [34]. Regarding the *in vitro*-based studies, we argue that the findings of these reports may overestimate the absolute requirement for Rac2 in various innate immune cell functions. While neutrophils and macrophages isolated from the peripheral blood and bone marrow of Rac2^−/−^ mice display significant defects in phagocytosis, chemotaxis and/or ROS production, we and others propose that these cells may not exhibit such severe deficiencies when present in the setting of an active immune-inflammatory environment. In this regard, neutrophils isolated from the peritoneum of Rac2^−/−^ mice following thioglycollate injection showed only marginal defects in ROS production and, similarly, bone marrow-derived Rac2^−/−^ neutrophils stimulated with tumour necrosis factor (TNF)-α showed only a modest impairment in ROS production following secondary stimulation [13]. Thus, in the context of activating conditions (be they cytokine stimulation, inflammation, etc.), the loss of Rac2 may be partially compensated for by, presumably, other Rac isoforms. It is important to acknowledge that studies have consistently observed marked, albeit incomplete, defects in neutrophil chemotaxis and migration both *in vitro* and *in vivo*, in mice and humans [11], [13], [14], [25]. Neutrophils are known to be a significant contributing cell type in the response mounted against *C. rodentium* [35], [36], although perhaps not as essential as some other immune cells. In the studies presented here, whatever impact the loss of Rac2 had on neutrophil migration was not sufficient enough to impair bacterial control/clearance.

Interestingly, despite the absence of *C. rodentium* in Rac2^−/−^ infected mice at day 22, we observed augmented colonic epithelial cell hyperplasia and exaggerated inflammation in the colon. These findings suggest that Rac2 may play a role, either directly or indirectly, in pathways related to the regulation and/or resolution of inflammation. That we documented dramatic increases in the production of IFN-γ and IL-17 from Rac2^−/−^ splenocytes recalled *in vitro* with *C. rodentium* lysate, further suggests that the regulatory defects may lie in the processes that influence T-cell responses.

Previous reports have demonstrated that Rac2 is involved in various T-cell processes including migration, differentiation and apoptosis [21], [24], [37], [38], and defects in any of these could have contributed to the heightened responses we observed in infected Rac2^−/−^ mice. The loss of Rac2 in T-cells may have affected development of regulatory T-cells and, hence, resulted in at least a partial loss of inflammatory regulation. The involvement of Rac2 in T-cell differentiation is not without precedent as some, although not all, studies have shown that Rac2 plays a role in Th1 cell differentiation [21], [24]. Although our results (i.e. IFN-γ production by splenocytes) do not support the idea of impaired Th1 development in infected Rac2^−/−^ mice, they do not rule out the possibility that defects may exist in the differentiation pathways of other T-cell phenotypes such as T regulatory cells. Although somewhat unlikely, it is possible that the exaggerated immune-responsiveness we report here is related to impaired T-cell migration to or within the colon. Rac2^−/−^ T-cells do exhibit chemotactic defects *in vitro* and Rac2^−/−^ mice have been reported to display abnormal peripheral T-cell distribution [21]. However, we did not observe any overt aggregations or limitations in overall T-cell distribution (sub-mucosa vs. mucosa) in the colons of infected Rac2^−/−^ mice. Alternatively, Rac2-deficiency may have impacted resolution mechanisms via some detriment to apoptosis. Along these lines, Rac2 has been shown to be a key signalling component of restimulation-induced cell death (RICD) [37]; a process that is thought to be an essential self-limiting negative feedback mechanism for controlling T-cell expansion during ongoing immune responses [39]. T-cells from Rac2^−/−^ mice and Rac2-siRNA treated human primary CD4^+^ T-cells displayed significantly impaired TCR-induced cell death [37]. Importantly, as RICD is thought to limit immunopathology that may arise from excessive effector T-cell expansion [39], it is plausible that defects in Rac2-mediated RICD may account for the exaggerated T-cell responses and associated inflammatory and hyperplasia we report here.

Our observations regarding *C. rodentium* infection in Nox2^−/−^ mice suggest that at least some of the heightened immunoresponsiveness we found in infected Rac2^−/−^ mice may not be a result of Rac2’s role in NADPH-derived ROS production. Interestingly, previous studies that have investigated the contribution of the Nox2 complex to host defense responses in the intestine have reported mixed results. Mice deficient in p47^phox^ displayed no overt differences compared to their WT controls in response to dextran sulphate sodium (DSS)-induced disease [40] and, interestingly, gp91^−/−^ mice were partially protected from DSS-induced responses [41]. On the other hand, a recent study reported that p40^phox−/−^ mice have increased inflammation and more severe colonic tissue injury in both DSS and Rag1^−/−^ mouse models of colitis [42]. The differences in these findings are somewhat difficult to reconcile strictly in terms of the role of Nox2-derived ROS in models of colitis, however, they may derive, in part, from the participation of ‘Nox’ components (i.e. p40^phox^, p47^phox^, gp91) in other, Nox2-independent, processes/signalling pathways.

A considerable body of research supports the notion that impairments in host immunity, be they related to inflammatory- or regulatory-associated networks, contribute to the pathogenesis of IBD. In the studies presented here, we sought to investigate whether a defect in RAC2, a well-recognized mediator of various fundamental innate and adaptive immune cell processes, may impact host immune responses in the intestine and the development of disease. Our findings demonstrate that Rac2-deficient mice subjected to an infection-based model of colitis develop worsened disease characterized by increased and protracted colonic epithelial cell hyperplasia and exacerbated colonic inflammation. Importantly, the findings presented here speak to the possible contribution of variants in the *RAC2* gene, which may affect RAC2 expression and/or function, on the development of intestinal disease in humans. The issue is of relevance as population-based genetic studies continue to implicate an increasing number of genes whose roles in the pathogenesis of IBD are not well understood. While many of these genes are thought to, individually, contribute only modestly to IBD risk, their identification will, at the very least, form the basis for more detailed analyses into the influence of gene pathways/networks. Coupled with detailed functional studies in both experimental models and patients, the knowledge gained is sure to furnish a foundation that not only inspires novel development but also guides the implementation of treatment strategies on a patient-by-patient basis.

## Supporting Information

Table S1 **Histological Score.**
(DOC)

Table S2 **Clinical Symptoms Score.**
(DOC)
