# Supplementary material for: Rac2-Deficiency Leads to Exacerbated and Protracted Colitis in Response to Citrobacter rodentium Infection
Source: PLoS One. 2013 Apr 16;8(4):e61629. doi: 10.1371/journal.pone.0061629 (PMC3628927; doi:10.1371/journal.pone.0061629)
Supplement: Table S1 — Histological Score. (DOC) [file pone.0061629.s001.doc]

Table S1. Histological Score

| **Criterion** | **Score**  **1** | **2** | **3** | **4** | **5** |
| --- | --- | --- | --- | --- | --- |
| Goblet Cells | --- | ↓ | ↓↓ | ↓↓↓ | ↓↓↓↓ |
| Mononuclear Cells – Mucosal | --- | ↑ | ↑↑ | ↑↑↑ | ↑↑↑↑ |
| Mononuclear Cells – Submucosal | --- | ↑ | ↑↑ | ↑↑↑ | ↑↑↑↑ |
| Polymorphonuclear Cells - Mucosal | --- | ↑ | ↑↑ | ↑↑↑ | ↑↑↑↑ |
