# Supplementary material for: Rac2-Deficiency Leads to Exacerbated and Protracted Colitis in Response to Citrobacter rodentium Infection
Source: PLoS One. 2013 Apr 16;8(4):e61629. doi: 10.1371/journal.pone.0061629 (PMC3628927; doi:10.1371/journal.pone.0061629)
Supplement: Table S2 — Clinical Symptoms Score. (DOC) [file pone.0061629.s002.doc]

Table S2. Clinical Symptoms Score

| **Criterion** | **Score**  **0** | **1** | **2** | **3** | **4** |
| --- | --- | --- | --- | --- | --- |
| Weight Loss (%) | None | 1-5 | 5-10 | 10-15 | >15 |
| Stool Consistency | Normal | Loose | Diahhrea |  |  |
| Appearance | Normal | Ruffled Coat | Hunched | Lethargic |  |
